# Identification of a Pyroptosis-Related Gene Signature for Predicting the Immune Status and Prognosis in Lung Adenocarcinoma

**DOI:** 10.3389/fbioe.2022.852734

**Published:** 2022-05-12

**Authors:** Zetian Gong, Qifan Li, Jian Yang, Pengpeng Zhang, Wei Sun, Qianhe Ren, Junjie Tang, Wei Wang, Hui Gong, Jun Li

**Affiliations:** ^1^ Department of Thoracic Surgery, The First Affiliated Hospital of Nanjing Medical University, Nanjing, China; ^2^ Department of Thoracic Surgery, The First Affiliated Hospital of Soochow University, Suzhou, China; ^3^ Department of Trauma Center, Affiliated Hospital of Nantong University, Nantong, China

**Keywords:** lung adenocarcinoma, pyroptosis, bioinformatics analysis, TCGA, PRKACA, GPX4, immune checkpoint genes

## Abstract

**Background:** Pyroptosis is a form of programmed cell death triggered by the rupture of cell membranes and the release of inflammatory substances; it is essential in the occurrence and development of cancer. A considerable number of studies have revealed that pyroptosis is closely associated to the biological process of several cancers. However, the role of pyroptosis in lung adenocarcinoma (LUAD) remains elusive. The purpose of this study was to explore the prognostic role of pyroptosis-related genes (PRGs) and their relationship with the tumor immune microenvironment (TIME) in LUAD.

**Methods:** Gene expression profiles and clinical information were downloaded from The Cancer Genome Atlas (TCGA) and Gene Expression Omnibus (GEO) databases. A prognostic PRG signature was established in the training set and verified in the validation sets. Functional enrichment and immune microenvironment analyses related to PRGs were performed and a nomogram based on the risk score and clinical characteristics was established. What is more, quantitative real-time PCR (qRT-PCR) analysis was applied in order to verify the potential biomarkers for LUAD.

**Results:** A prognostic signature based on five PRGs was constructed to separate LUAD patients into two risk groups. Patients in the high-risk group had worse prognoses than those in the low-risk group. The signature was identified as independent via Cox regression analyses and obtained the largest area under the curve (AUC = 0.677) in the receiver operating characteristic (ROC). Functional enrichment and immune microenvironment analyses demonstrated that the immune status was significantly different in the two subgroups and that immunotherapy may be effective for the high-risk group. Furthermore, qRT-PCR analysis verified that serum PRKACA and GPX4 could serve as diagnostic biomarkers for LUAD.

**Conclusion:** Overall, a risk signature based on five PRGs was generated, providing a novel perspective on the determinants of prognosis and survival in LUAD, as well as a basis for the development of individualized regimes.

## 1 Introduction

According to the latest global cancer statistics, lung cancers remain one of the most commonly diagnosed cancers, and they have the highest incidence of deaths worldwide (Sung et al., 2021). Lung adenocarcinoma (LUAD) is the most common histological subtype of non-small cell lung cancer (NSCLC), which also occupies almost 80% of lung cancer cases (Gridelli et al., 2015). Despite progress in surgery, targeted therapy, chemotherapy, and radiotherapy, the 5-year overall survival (OS) rate for lung cancer remains only around 21% (Siegel et al., 2021). Currently, therapeutic regimens for individual LUAD patients are based mainly on specific factors such as radiomic features, tumor-node-metastasis (TNM) staging, tumor subtypes, and the differentiation grade. With the rapid rise in precision medicines, novel therapeutic schedules, especially immunotherapies and targeted therapies, have been proposed to prolong the lives of LUAD patients (Bronte et al., 2010; Saito et al., 2018). However, only a portion of patients have received benefits from them, leaving an urgent need to explore potential biomarkers for efficient and prognostic predictions.

Pyroptosis, also known as inflammatory “necrosis,” is an inflammatory caspase-dependent cell death type triggered by the cell rupture and the release of many proinflammatory factors, including IL-1β, IL-18, ATP, and HMGB1 (Fang et al., 2020; Tang et al., 2020). It has been demonstrated that the process of pyroptotic cell death is mediated mainly through GSDMD (gasdermin D)-dependent activation regulated by caspase 1/4/5/11 (Shi et al., 2015). Activated caspases cleave the hinge region between the N- and C-terminal domains of GSDMD, releasing the segment with lethal activity and leading to pyroptosis (Ding et al., 2016). Several studies have indicated that pyroptosis was both a friend and a foe of cancers (Nagarajan et al., 2019; Xia et al., 2019; Fang et al., 2020). On the one hand, the inflammatory mediators released and several signaling pathways are bound up with the tumorigenesis and their chemotherapeutic drugs resistance. On the other hand, as a type of programmed cell death, pyroptosis can suppress the emergence and progression of tumors. In NSCLC, a high level of GSDMD expression was shown to be linked with invasive features, including more advanced TNM stages and larger tumor sizes (Burdette et al., 2021). Recent studies have identified pyroptosis-related gene (PRG) signatures for the prognosis of ovarian cancer and gastric cancer (Shao et al., 2021; Ye et al., 2021), while the performance of PRGs in LUAD has not yet been clarified.

Given the existing findings, we know that pyroptosis is critical to the development of tumors and to antitumor processes; however, its precise functions in LUAD have not been explored as extensively. In the present work, we aimed to construct a scoring model based on PRGs to predict the prognosis of LUAD and explore the latter’s relationship with immune checkpoint genes (ICGs), hoping to find additional therapeutic targets.

## 2 Materials and Methods

### 2.1 Data Acquisition and Processing

The lung adenocarcinoma RNA-seq (FPKM) data and the corresponding clinical information were obtained from the TCGA database (https://portal.gdc.cancer.gov/). The cohort consisted of 497 tumor tissues and 54 normal tissues, with the complete clinical information of 486 patients (tumor = 439, normal = 47) extracted as a training set. The Ensemble IDs were transformed into gene symbols via the use of the “rtracklayer” and “dplyr” R packages, and the pieces of clinical information were merged into a single matrix for further analysis. To increase the reliability of the study, two Gene Expression Omnibus (GEO) datasets, i.e., GSE31210 and GSE50081 (both using the GPL570 platform), which contained the microarray-based expression data of LUAD patients and the relevant clinical information (n = 536), were extracted for validation from the GEO website (https://www.ncbi.nlm.nih.gov/geo/). In this study, we also identified 79 ICGs from a review of the literature (Pardoll, 2012; Hu et al., 2021), most of which were ligands, receptors or important molecules in immune checkpoint pathways (Supplementary Table S1).

### 2.2 Identification of Differentially Expressed Pyroptosis-Related mRNAs

A total of 33 PRGs were extracted from prior reviews (Ye et al., 2021) and are presented in Supplementary Table S2. Differentially expressed PRGs (DE-PRGs) were identified in the training cohort between normal and tumor tissues, using the “limma” R package with thresholds of *p* < 0.05.

### 2.3 Establishment and Validation of the Pyroptosis-Related Prognostic Gene Signature

To identify the prognostic genes among all PRGs, we further employed Cox regression analysis with the “survival” R package to assess the links between each gene and survival status in the training cohort. To avoid omissions, we set 0.2 as the cut-off *p*-value, and seven survival-related genes were screened for further analysis. Subsequently, multivariate Cox regression analysis was conducted to narrow down the candidate genes based on the lowest Akaike information criterion (AIC). Ultimately, five PRGs and their coefficients were retained. A prognostic risk score was created for each patient via the following formula: Risk score = ∑Coef (PRGs) * Exp (PRGs), where Exp (PRGs) is the relative expression of the candidate PRGs, and Coef (PRGs) is the regression coefficient. Based on the median value of the risk score, patients in the training set were divided into the high-risk group and the low-risk group. The OS between the two groups was compared by means of Kaplan–Meier analysis with the “survival” and “survminer” R packages. The predictive performance of the model was further validated in two GEO datasets (GSE31210 and GSE50081). Samples in the validation cohort were separated into high-risk and low-risk groups based on the formula for the risk score derived from the training dataset, respectively. The receiver operating characteristic (ROC) curve was used to assess the prognostic performance through the “timeROC” R package. The area under the curve (AUC) of each cohort was calculated for detailed evaluations.

### 2.4 Independent Prognostic Analysis of the Risk Score

We extracted the clinical information (age, gender, AJCC stage, TNM stage, tobacco history, and anatomical location) of patients in the training cohort. These elements were analyzed in company with the risk score in our regression model, employing univariate and multivariable Cox regression models. Furthermore, a time-dependent ROC curve was used to evaluate the predictive accuracy for OS by different clinicopathological factors and risk scores by means of the “survivalROC” package.

### 2.5 Development of a Predictive Nomogram

A nomogram incorporating the signature and clinical parameters was developed via the “rms” R package to predict the overall survival of LUAD patients. Then, the calibration curves and ROC curves were plotted to assess the predictive accuracy of the nomogram.

### 2.6 Functional Enrichment Analysis of PRGs

Depending on the median risk score, patients in the training cohort were stratified into two subgroups. The differentially expressed genes (DEGs) between the low- and high-risk groups were filtered at the specific threshold (|log2FC| ≥ 1 and FDR < 0.05). To clarify the biological functions of the prediction model, gene ontology (GO) and Kyoto Encyclopedia of Genes and Genomes (KEGG) enrichment analyses were performed, based on the DEGs, by applying the “clusterProfiler” R package with the criteria of *p* < 0.05 and FDR < 0.05. The “gsva” R package was also employed to conduct the ssGSEA to calculate the scores of infiltrating immune cells and to assess the activity of immune-related pathways.

### 2.7 Evaluation of the Immune Status Between the Two Subgroups

To further explore the link between the prediction model and the immune system, the single-sample gene set enrichment analysis (ssGSEA) method was utilized to quantify the overall immune status of the two subgroups by analyzing the expression profiles of the 29 immune signature gene sets. Subsequently, the ESTIMATE algorithm was performed to calculate stromal and immune scores, determining the levels of stromal and immune cell tumor infiltration. Thereafter, correlations between the risk score and several key ICGs, such as PD-L1, CTLA4, LAG-3, and so on, were evaluated. Spearman correlation analyses were used to examine the relationship among the risk score, the stromal and immune scores, and the expression of ICGs.

### 2.8 Protein Levels of NRGs in the HPA Database

The Human Protein Atlas (HPA) is a database containing all of the human proteins in cells, tissues and organs, where all images of tissues are stained via immunohistochemistry. To compare the protein expression levels related to the prognostic signature, we extracted the immunohistochemical images of the candidate PRGs from the HPA database (https://www.proteinatlas.org/).

### 2.9 Cell Lines and Cell Culture

Three LUAD cells—namely A549, H1299, and H1650—and one normal epithelial cell line (HBE) were purchased from the Cell Bank of the Chinese Academy of Sciences (Shanghai, China) and cultured in an RPMI-1640 medium (HyClone, Logan, UT, United States) with 10% fetal bovine serum at 37°C in a humidified atmosphere with 5% CO2.

### 2.10 Real-Time Quantitative Reverse-Transcriptase Polymerase Chain Reaction Analysis

Total RNA was extracted by TRIzol reagent (Thermo Fisher Scientific, Carlsbad, CA, United States) according to the protocol and reverse-transcribed to cDNA through the use of random primer amplification. Real-time qRT-PCR analysis was carried out using Platinum SYBR Green qPCR SuperMix-UDG kits (Life Technologies, Gaithersburg, MD, United States). Primers used for the qRT-PCR analysis were performed as follows. Glyceraldehyde 3-phosphate dehydrogenase (GADPH) levels were used to normalize PRKACA and GPX4 expression. Relative expression was calculated using the ∆∆Ct method.

**Table:** 

Gene	Forward primer	Reverse primer
GAPDH	ACA​ACT​TTG​GTA​TCG​TGG​AAG​G	GCC​ATC​ACG​CCA​CAG​TTT​C
PRKACA	CAA​GGA​GAC​CGG​GAA​CCA​CTA	CAT​TCA​GGG​TGT​GTT​CGA​TCT​G
GPX4	GAG​GCA​AGA​CCG​AAG​TAA​ACT​AC	CCG​AAC​TGG​TTA​CAC​GGG​AA

### Statistical Analysis

**FIGURE 1:**
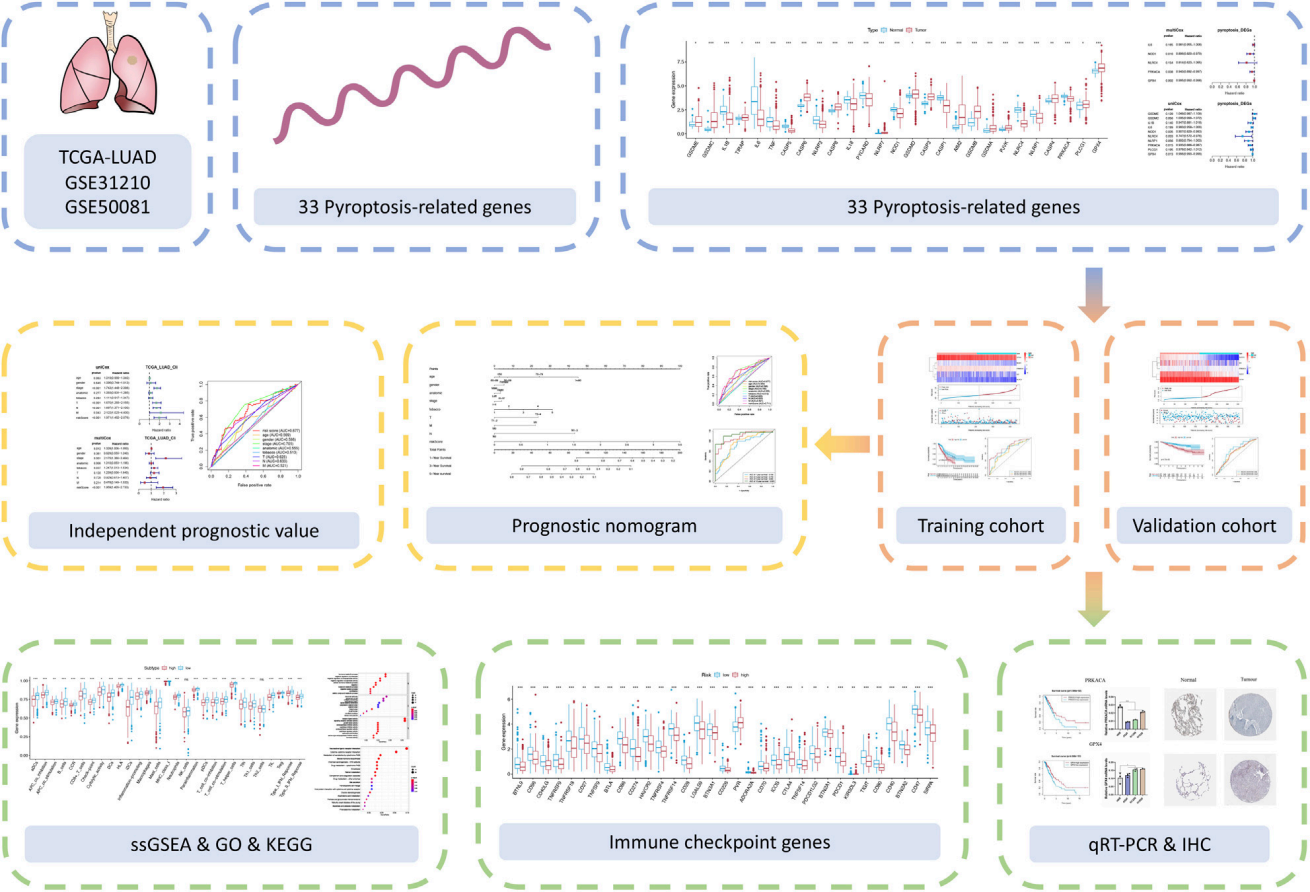
The flowchart of the study.

All statistical analyses were performed using R language (Version 4.1.0). The Kaplan–Meier method with a two-sided log-rank test was performed to compare the OS of patients between the two subgroups. To determine the independent risk characteristics, univariate and multivariate Cox analyses were applied. Correlation coefficients between two non-bivariate normally distributed variables were computed via Spearman analyses. The hazard ratios (HRs) and the 95% confidence intervals of the aforementioned elements were estimated in order to quantify the strength of these associations. All statistical tests were two-tailed. The overall flowchart is shown in Figure 1.

## 3 Results

### 3.1 Defining the Differentially Expressed PRGs in LUAD

**FIGURE 2:**
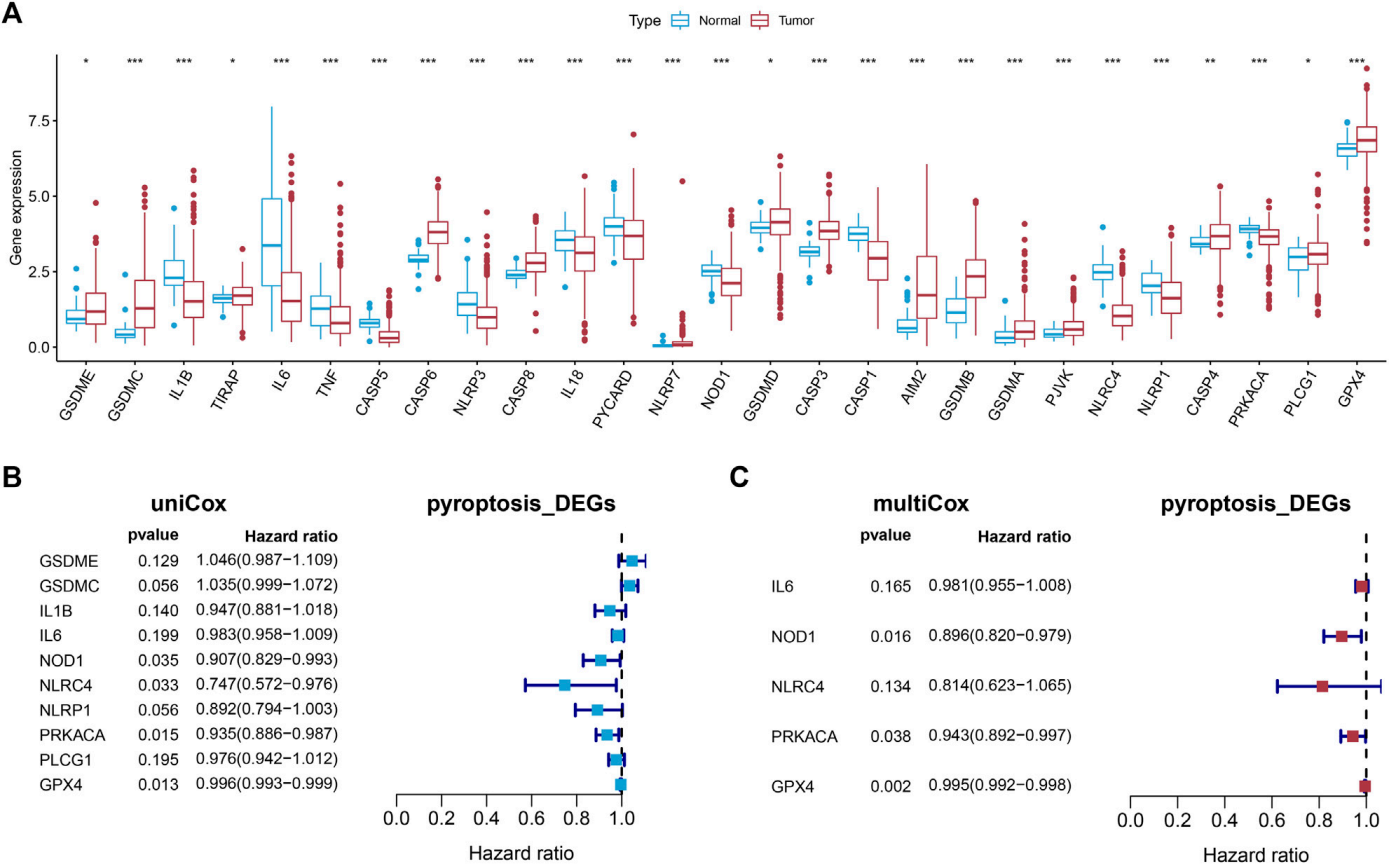
Establishment of the prognostic model. **(A)** The boxplot showed the differentially expressed PRGs between normal and tumor tissues of LUAD. **(B,C)** Univariate and multivariate Cox regression identified the PRGs associated with prognosis.

The expression of the 33 PRGs in LUAD and normal lung tissues was first obtained by means of the TCGA dataset. Following differential expression analysis of the training set, 27 PRGs were either upregulated or downregulated in LUAD. More definitely, the expression of IL6, NLRC4, CASP5, IL1B, CASP1, NLRP3, NLRP1, PYCARD, IL18, PRKACA, TNF, and NOD1 was raised, while the expression of AIM2, TIRAP, PLCG1, GSDMD, CASP4, GPX4, CASP8, GSDME, PJVK, CASP3, CASP6, GSDMA, GSDMB, NLRP7, and GSDMC was declined in LUAD in comparison with normal tissues (Figure 2A, FDR < 0.05).

### 3.2 The Establishment and Verification of a Pyroptosis-Related Prognostic Model

**FIGURE 3:**
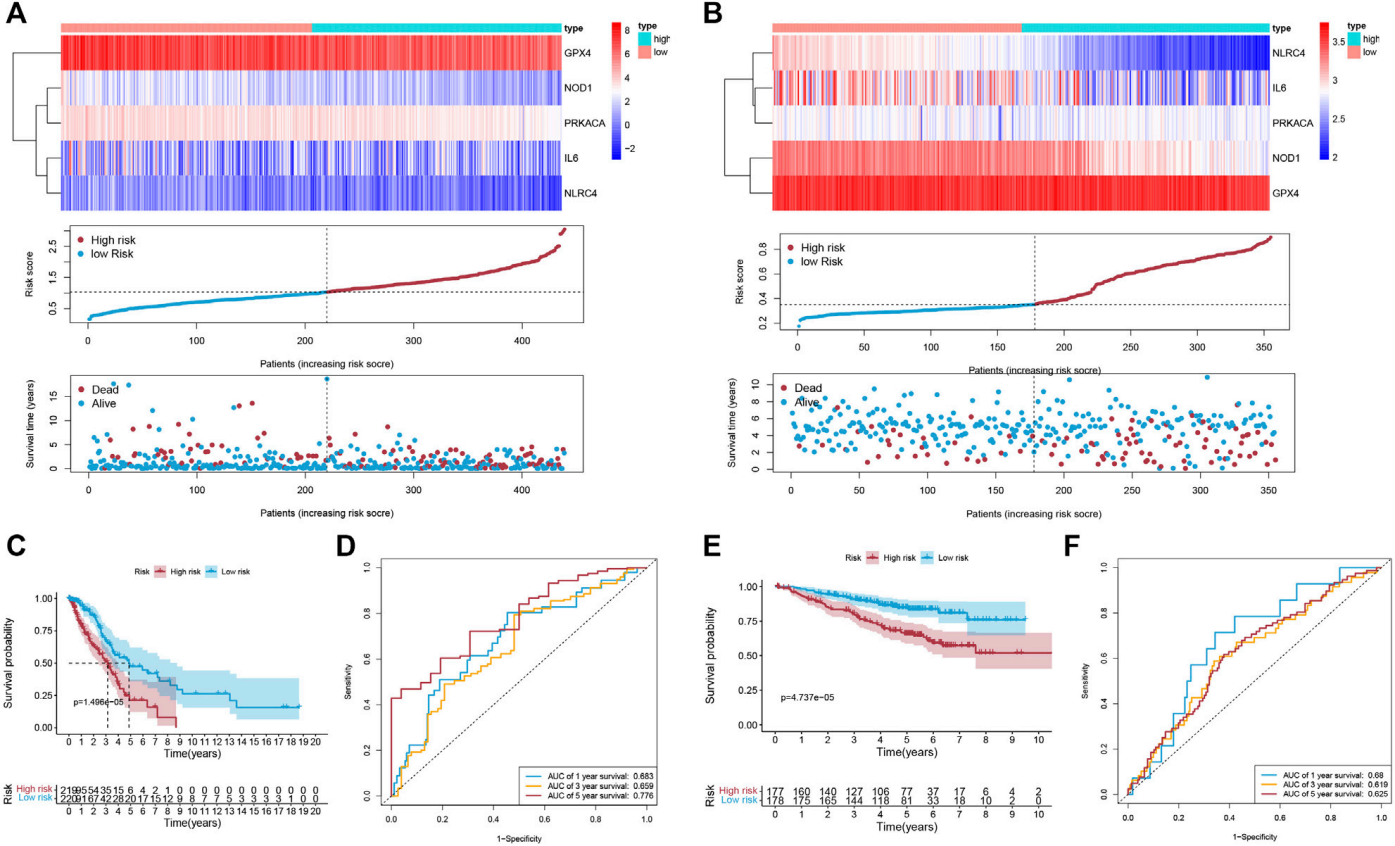
The accuracy of the prognostic model. **(A,B)** The gene expression pattern; distribution of risk scores between the two groups; distribution of survival status in the training cohort **(A)** and validation cohort **(B)**. **(C,E)** K-M survival curves of OS in the training cohort (C; *p* = 1.496e-05) and validation cohort (E; *p* = 4.737e-05). **(D,F)** The ROC curve representing patients’ survival for different numbers of years in the two cohorts, AUC of 1-year, 3-year, and 5-year OS were 0.683, 0.659, and 0.776 (respectively) in the training cohort **(D)**, and were 0.68, 0.619, and 0.625 (respectively) in the validation cohort **(F)**.

Univariate Cox regression analysis was first applied in order to verify the candidate PRGs associated with prognosis (*p* < 0.2) (Figure 2B), and multivariate Cox regression analysis further identified five PRGs, namely IL6, NOD1, NLRC4, PRKACA, and GPX4, based on the lowest AIC (1,059.83) (Figure 2C). The formula is shown as follows: risk score = (−0.019 * expression level of IL6) + (−0.110 * expression level of NOD1) + (−0.205 * expression level of NLRC4) + (−0.059 * expression level of PRKACA) + (−0.005 * expression level of GPX4). We classified the LUAD cases into low-risk (n = 220) and high-risk (n = 219) groups depending on the median risk score. The risk score distribution, survival status, and gene expression pattern of the two groups are presented in Figure 3A. As the risk score raised, the patients’ risk of death also increased and the survival time reduced. The Kaplan–Meier analysis revealed that LUAD patients in the high-risk group had shorter OS (Figure 3C, *p* = 1.496e-05), with AUCs of 0.683, 0.659, and 0.776 in the 1-year, 3-year, and 5-year ROC curves, respectively (Figure 3D).

### 3.3 Validation of the Signature in Two GEO Datasets

To evaluate the accuracy and stability of the prognostic signature, two GEO datasets (GSE31210 and GSE50081, both based on GPL570, Supplementary Table S3) were performed as external validations. Patients in the validation cohort were classified into low-risk and high-risk groups depending on the formula for the risk score derived from the training cohort, respectively (Figure 3B). Similarly, in the validation cohort, better OS belonged to the patients with low-risk scores (Figure 3E, *p* = 4.737e-05), with AUCs of 0.68, 0.619, and 0.625 in the 1-year, 3-year, and 5-year ROC curves, respectively (Figure 3F).

### 3.4 Risk Factors Predictive of Survival in LUAD

**FIGURE 4:**
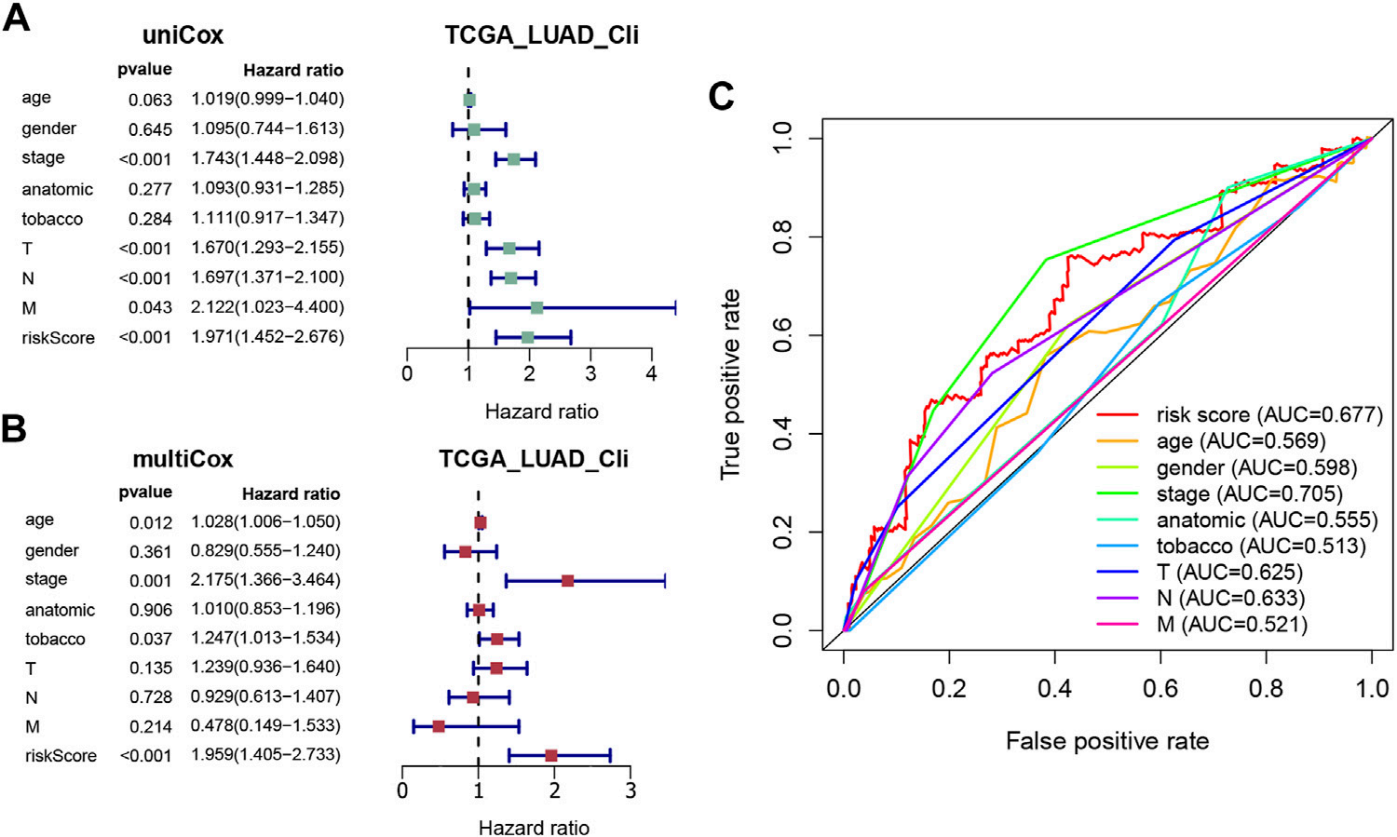
Evaluation of the prognostic accuracy of the model and other clinicopathological characteristics. **(A,B)** Univariate and multivariate Cox regression analyses of the risk score and other clinical parameters (age, gender, AJCC stage, anatomic location, tobacco history, T, N, and M). **(C)** ROC curves for the risk score (AUC = 0.677) and other clinical features.

Univariate and multivariate Cox regression analyses were applied to evaluate whether the risk score derived from the signature could function as an independent prognostic factor, using the “survival” package. As shown in Figure 4A, the AJCC stage (*p* < 0.001), T stage (*p* < 0.001), N stage (*p* < 0.001), M stage (*p* = 0.043), and risk score (*p* < 0.001) were significantly related to OS in the univariate Cox regression analysis, with only the AJCC stage (*p* = 0.001) and risk score (*p* < 0.001) also being significantly related to OS in the multivariate Cox regression analysis (Figure 4B). Furthermore, a time-dependent ROC curve was performed to testify as to the predictive accuracy. According to the results, the AUC of the risk score was 0.677, which was higher than the AUC of the T stage, N stage, and M stage and similar to the AUC of the AJCC stage (Figure 4C), indicating that the prognostic risk model was relatively reliable. To sum up, the prediction model could be regarded as an independent prognostic factor for LUAD patients.

### 3.5 Construction of a Predictive Nomogram

**FIGURE 5:**
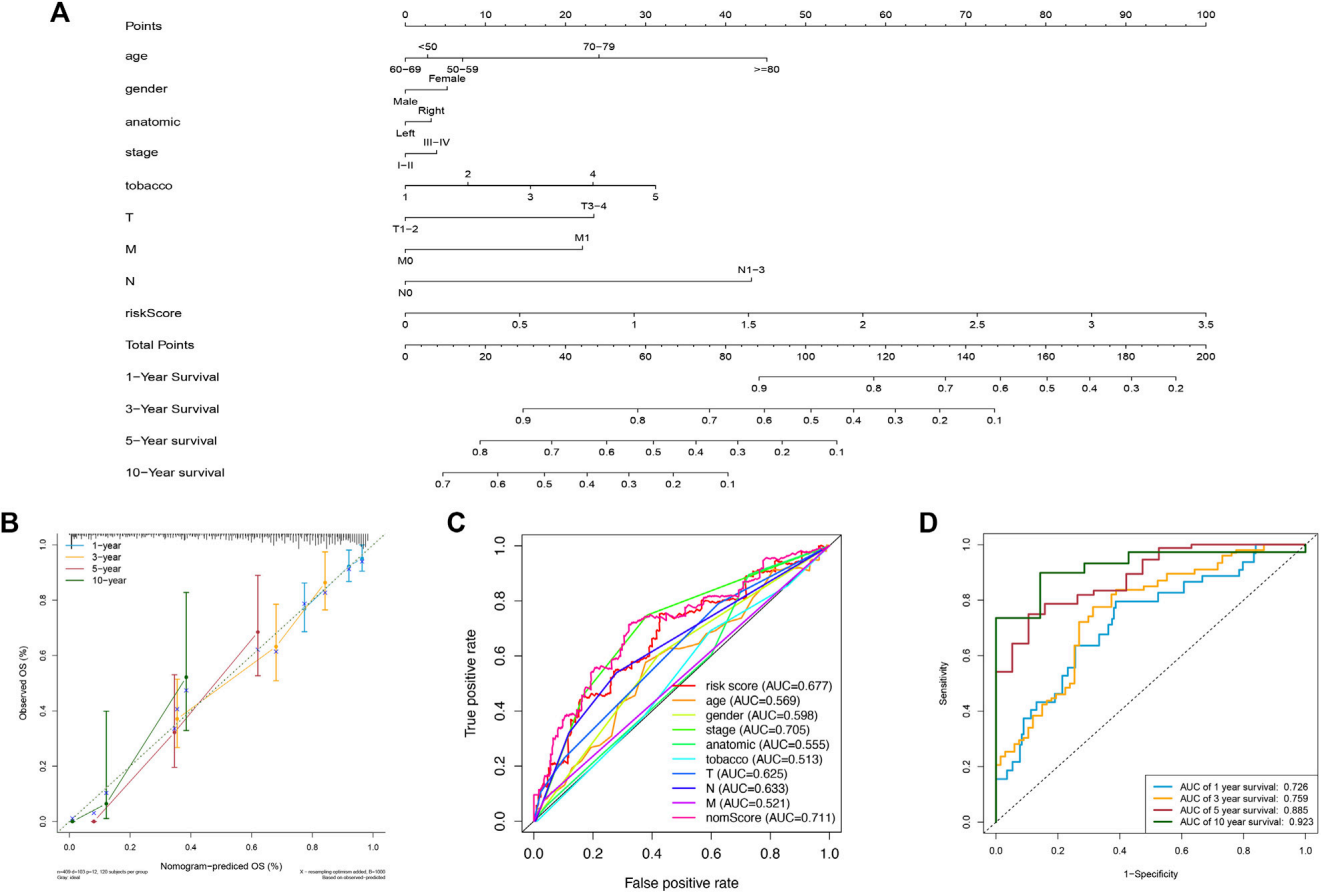
Construction and assessment of a nomogram. **(A)** The nomogram of 1-year, 3-year, 5-year, and 10-year OS on basis of the risk model and other clinical features. **(B)** Calibration plots used for evaluating the consistency between the actual and the predicted 1-year, 3-year, 5-year, and 10-year OS. **(C)** ROC curves for the nomscore (AUC = 0.711) and other elements. **(D)** The ROC curves of nomscore for predicting OS. The AUC of 1-year, 3-year, 5-year, and 10-year OS were 0.726, 0.759, 0.885, and 0.923 respectively.

To predict the patients’ survival time accurately, nomograms are usually applied by calculating the nomogram score based on each prognostic elements included in the nomogram (Balachandran et al., 2015). In this study, we established a nomogram to evaluate the probabilities of 1-year, 3-year, 5-year, and 10-year survival by using the risk score and other clinicopathological elements, like gender, AJCC stage, TNM stage, tobacco history, and anatomical location (Figure 5A). Calibration curves were also plotted and showed a high degree of consistency between the actual and the predicted 1-year, 3-year, 5-year, and 10-year survival when compared to the reference line (Figure 5B). Then, we observed that the AUC of the nomscore calculated from the nomogram was 0.711, which was greater than the riskscore (AUC = 0.677) (Figure 5C). Furthermore, the AUC of the nomscore in the 1-year, 3-year, 5-year, and 10-year ROC curves reached 0.726, 0.759, 0.885, and 0.923 (Figure 5D). These results suggested that the prediction efficiency would be more accurate and reliable when the risk score was jointed with other clinicopathological parameters.

### 3.6 Functional Enrichment Analysis

**FIGURE 6:**
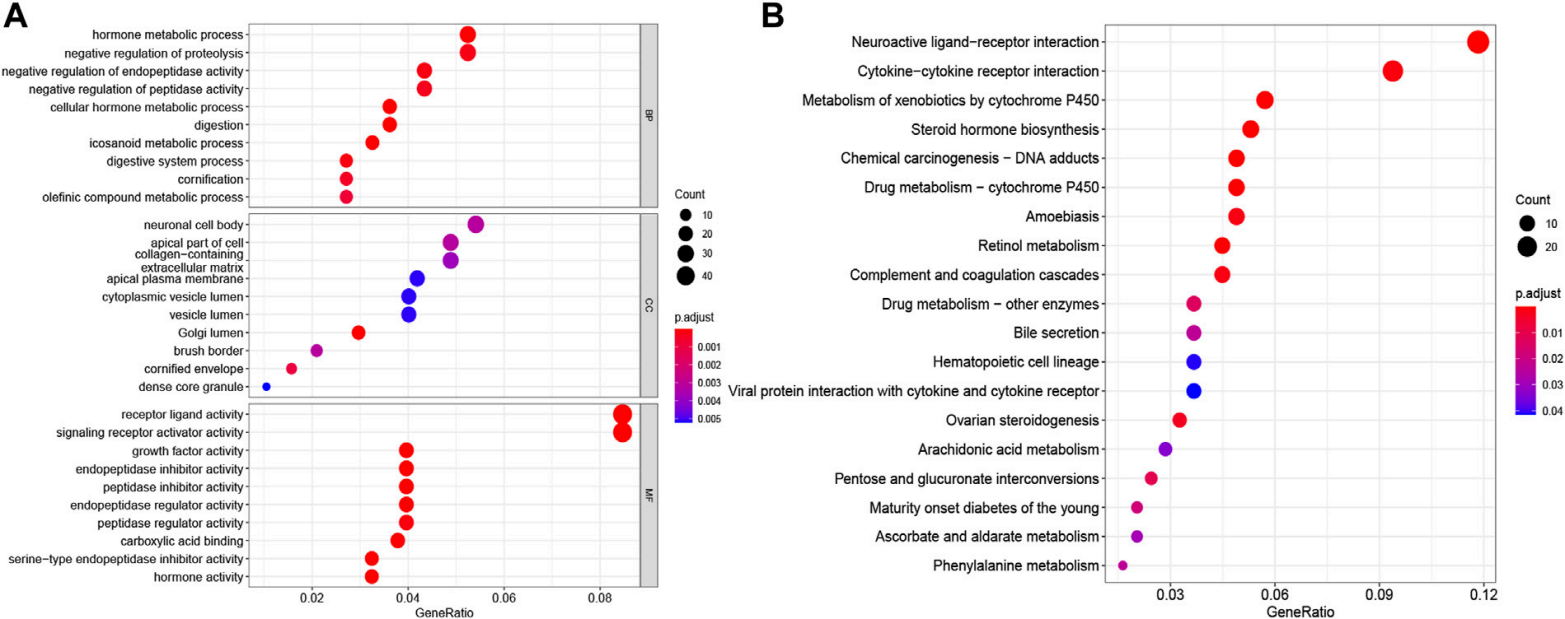
Functional enrichment analyses depending on the signature. The bubble graphs for GO enrichment analysis **(A)** and KEGG pathway analysis **(B)** were displayed. The bigger the bubble, the richer the genes, and the darker the red, the more pronounced the difference.

To further elucidate the biological functions and pathways of DE-PRGs in pyroptosis, the “limma” R package was applied in order to extract the DEGs between the two groups. FDR < 0.05 and |log2FC | ≥ 1 were statistically significant. Altogether, 820 DEGs were identified in the TCGA cohort. Among them, 343 genes were highly expressed in the high-risk group, while the other 477 genes were low expressed. Subsequently, based on the DEGs, GO enrichment analysis and KEGG pathway analysis were performed. The top GO terms were “hormone metabolic process” in the biological process (BP), “neuronal cell body” in the cellular component (CC), and “receptor ligand activity” in the molecular function (MF), respectively (Figure 6A). According to KEGG analysis, “neuroactive ligand−receptor interaction,” “cytokine−cytokine receptor interaction,” “steroid hormone biosynthesis,” and “chemical carcinogenesis—DNA adducts” were the main pathways (Figure 6B). On the whole, the results implied that the DEGs were mainly correlated with the immune response, receptor interaction, and chemical metabolism.

### 3.7 Comparison of the Tumor Immune Microenvironment Between Groups

**FIGURE 7:**
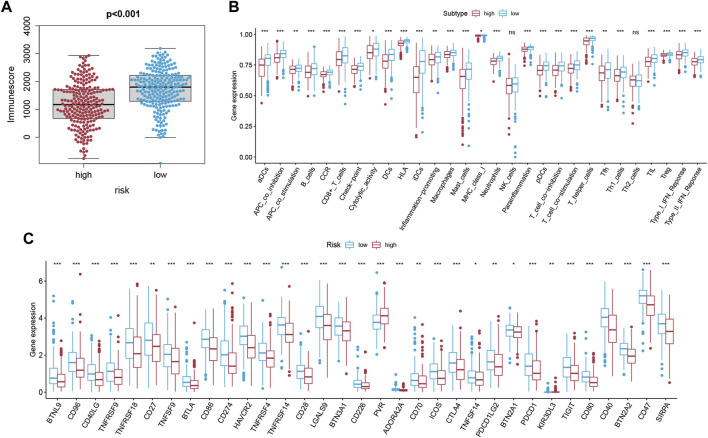
Analysis of the immune status of LUAD. **(A)** The correlation between the risk score and the immune score was displayed (*p* < 0.001). **(B)** Differential distribution of enrichment scores of 16 immune cells types and 13 immune-related pathways between the low-risk (blue box) and high-risk (red box) groups via ssGSEA. **(C)** Boxplots of the expression level of ICGs between the two subgroups (low-risk: blue; high-risk: red). Only the expression level of PVR was positively correlated to the risk score.

**FIGURE 8:**
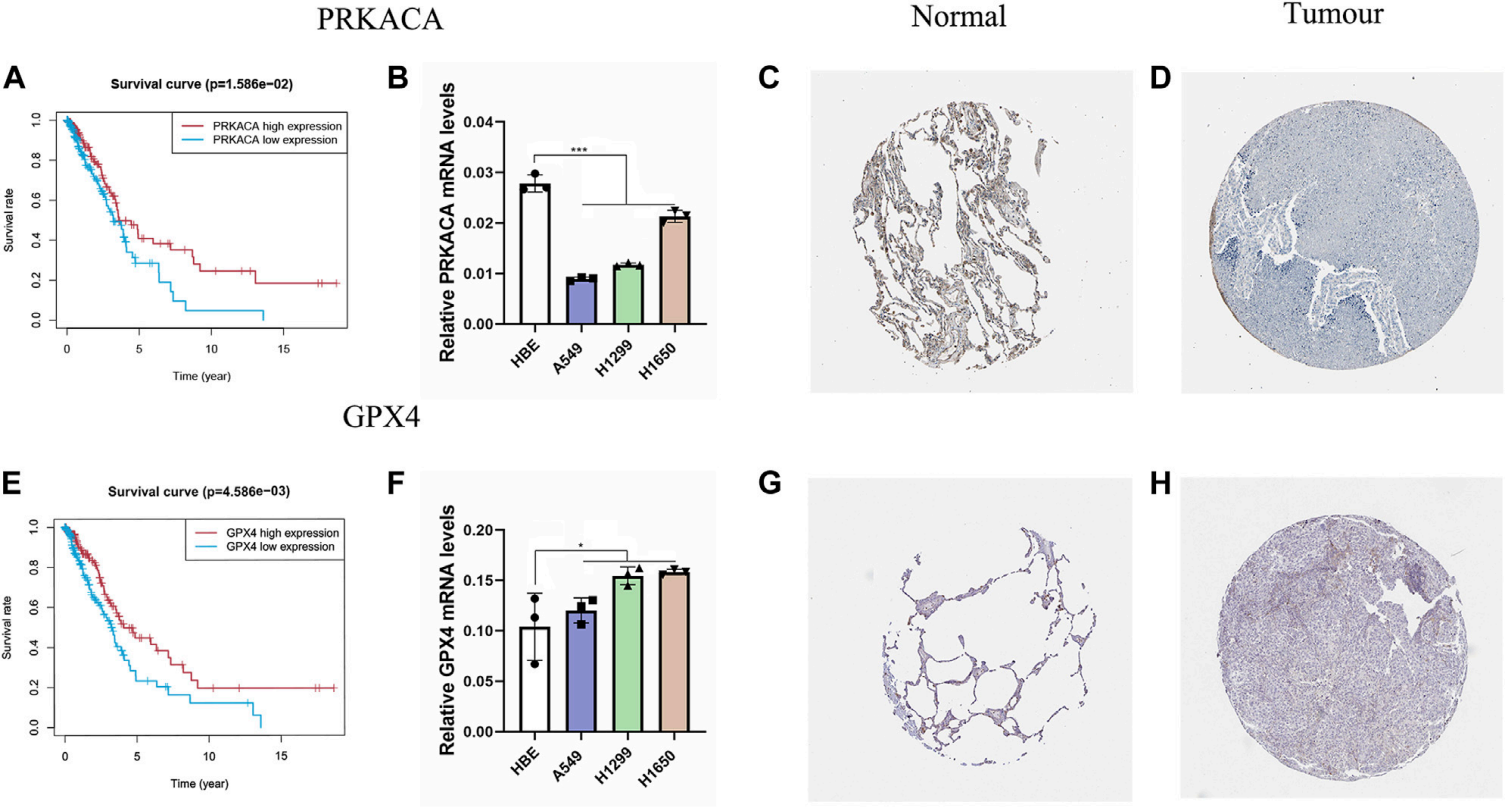
Validation of PRKACA and GPX4. **(A,E)** The prognostic differences of PRKACA (*p* = 1.586e-02) and GPX4 (*p* = 4.586e-03) between the high-risk and low-risk groups were investigated. **(B,F)** Relative mRNA levels of PRKACA and GPX4 were displayed via qRT-PCR. PRKACA was highly expressed in normal tissues, and GPX4 showed the opposite. **(C–D,G–H)** Protein expression of the two candidate genes in the HPA database. **(C–D)** Protein expression of PRKACA. **(G–H)** Protein expression of GPX4.

Based on the ESTIMATE algorithm, we successfully obtained the immune scores, stromal scores, and estimate scores. The immune scores were distributed between −934.47 and 3,190.06 and represented a significant difference between the subgroups (Figure 7A, *p* < 0.001). Thereafter, we applied ssGSEA to quantify the immune activation level between the two subgroups by analyzing the expression profiles of the 29 immune signature gene sets. As shown in Figure 7B, the levels of immune cell infiltration, especially of B cells, CD8^+^ T cells, neutrophils, T helper 1 (Th1) cells, tumor-infiltrating lymphocytes (TILs), and regulatory T (Treg) cells, were generally lower in the high-risk group than in the low-risk group. Moreover, the levels of 13 immune pathways displayed a similar distribution between the two groups. Furthermore, from the previous literature, 79 ICGs were extracted (Supplementary Table S1). After removing the HLA related genes, 60 genes remained, in which 51 out of 60 ICGs have expression values. The results of the Spearman correlation analyses between the risk score and the ICGs revealed that the signature may be closely related to immunotherapy. As shown in Figure 7C, most of the ICGs were highly expressed in the low-risk group, except for PVR, which may be a promising therapeutic target. We then validated our results through the use of clinical specimens from the HPA. The OS and the histological expressions of PRKACA and GPX4 in normal and tumor tissues were exhibited, in accordance with the results front (Figures 8A,C–E,G,H).

### 3.8 qRT-PCR Analysis

We validated this result by applying the qRT-PCR analysis to three LUAD cell lines and a normal lung cell line. We found that the mRNA expression level of PRKACA was higher in the normal cell line than in the cancer cell lines, and for GPX4 it was the opposite, consistent with our results above (Figures 8B,F).

## 4 Discussion

Lung cancer is one of the leading causes of cancer morbidities and the most common cause of cancer-related deaths worldwide (Sung et al., 2021). For early-stage LUAD patients, surgery is the recommended treatment (Vansteenkiste et al., 2014), whereas chemotherapy, radiotherapy, immunotherapy, and targeted therapy are recommended for advanced LUAD patients (Hirsch et al., 2017). Several studies have demonstrated a strong association between TMB and the clinical benefits of receiving immunotherapy (Proto et al., 2019; Samstein et al., 2019). Moreover, TIME is closely correlated with the efficacy of immunotherapy (Frankel et al., 2017; Lei et al., 2020), suggesting that immunotherapy may be more effective for high-risk patients based on the risk model.

Pyroptosis is a type of programmed cell death triggered by a family of inflammatory caspases that plays as a double-edged sword in the tumorigenesis and therapeutic mechanisms, which has been newly recognized in recent years. Pyroptosis was found to release inflammatory factors and stimulate normal cells, leading to transformation into tumor cells (Karki and Kanneganti, 2019). Having said that, pyroptosis can also promote tumor cell death and restrain proliferation and migration of cancer cells, making pyrolysis itself a hopeful prognostic and therapeutic target for cancer (Zheng and Li, 2020). A pyroptosis-related signature has been established to predict prognosis in ovarian cancer (Ye et al., 2021). While, the role of PRGs in LUAD has not yet been identified; thus, our study aimed to explain it.

In the present study, 33 PRGs were systematically analyzed to identify those associated with OS. Following the differential expression analysis, univariate Cox regression analysis, and multivariate Cox regression analysis, five optimal mRNAs, namely IL6, NOD1, NLRC4, PRKACA, and GPX4, were screened out for the pyroptosis-related prognostic signature. In our study, GPX4 and PRKACA were found to be elements of the prediction model. Previous literature indicated that GPX4, an antioxidant enzyme that participate in repairing oxidative damage to lipids, was an important negative regulator of the pyroptotic cell death pathway (Kang et al., 2018; Russo and Rathinam, 2018; Kajarabille and Latunde -Dada, 2019). PAKACA is a catalytic subunit alpha of protein kinase A activated by cAMP that closely related to the progression of tumor (Chao et al., 2021). Increased transcription of PRKACA has been detected in patients of breast cancer that resistant to trastuzumab, which becomes a routine treatment for HER2-positive breast cancer (Moody et al., 2015). This suggests that PRKACA may perform as a biomarker for cancer and a prognostic indicator. The five PRGs’ signature was testified to be an independent indicator for LUAD prognosis. Following this, a prediction model depending on the PRGs’ signature was constructed. The AUC of the signature could reach 0.776 in the training set when predicting 5-year survival.

Until now, the mechanism of pyroptosis has not been fully discovered. What we know is that as tumors progress, multiple cell death modes may be concurrent and interact with one another (Fritsch et al., 2019). Generally, pyroptosis features the release of many proinflammatory factors and the rupture of cell plasma membranes (Zheng and Li, 2020). We analyzed the DEGs between two subgroups and discovered they were mainly involved in receptor ligand activity and metabolic processes, implying that dying cells may induce complex metabolic processes. According to the GO and KEGG analyses results, we have reason to suppose that pyroptosis plays a vital role in the regulation of the tumor microenvironment.

Cancer immunotherapies targeting immune checkpoints have been proven to improve OS in various cancers (Dyck and Mills, 2017; Li et al., 2019). Thus, exploring novel targets and developing new schemes for antitumor therapy are always main tasks within current medicine. In this study, we hoped to discover the association between the signature and TIME. Based on the ESTIMATE algorithm and ssGSEA, we could speculate that in the high-risk group there is an overall lesion of immune functions. Previous researches revealed that Treg cells were recruited into the human tumor microenvironment and inhibited T cell immunity to abolish the therapeutic efficacy of PD-L1, CTLA-4, and the TGF-β blockade, regardless of whether they were live or apoptotic (Zou, 2006; Maj et al., 2017; Principe et al., 2021). Surprisingly, the level of Treg cells was higher in the low-risk group than in the high-risk group, which indicated that immunotherapy may be effective for the high-risk group. Furthermore, we detected the correlation between the risk score and the expression levels of ICGs. The results demonstrated that the level of poliovirus receptor (PVR, CD155) was higher in the high-risk group. PVR, a member of the nectin-like family of adhesion molecules, has been proved to decrease the expansion and function of tumor antigen-specific CD8^+^ T cells. PVR also has a high affinity to TIGHT, which is a promising new target for cancer immunotherapy (Shibuya et al., 1996; Kucan Brlic et al., 2019; Chauvin and Zarour, 2020). According to these findings, immunotherapy based on PVR may be promising for LUAD patients applicable to the model.

Despite the prognostic value of the signature, this study still encountered several limitations which must be considered. First, our report was retrospective and based on public databases, devoid of certain crucial clinicopathological information. Second, the way in which pyroptosis modulates the precise process of LUAD is unclear. Moreover, further biochemical experiments, such as immunohistochemistry, cell function experiments, and so on, need to be conducted to confirm the findings.

## 5 Conclusion

In conclusion, a prognostic signature of five PRGs was established in LUAD and validated in the GEO datasets to explore the role of pyroptosis in tumor malignancy. These PRGs were also associated with TIME, as well as helping to predict potential therapeutic regimens for LUAD. Further studies are necessary in order to verify these results in our study.

## Data Availability

The original contributions presented in the study are included in the article/Supplementary Material, further inquiries can be directed to the corresponding authors.
